# DNA binding by the antimalarial compound artemisinin

**DOI:** 10.1038/s41598-021-03958-6

**Published:** 2022-01-07

**Authors:** Sladjana Slavkovic, Aron A. Shoara, Zachary R. Churcher, Elise Daems, Karolien de Wael, Frank Sobott, Philip E. Johnson

**Affiliations:** 1 Department of Chemistry and Centre for Research on Biomolecular Interactions, York University, 4700 Keele St., Toronto, ON M3J 1P3 Canada; 2 BAMS Research Group, University of Antwerp, Groenenborgerlaan 171, 2020 Antwerp, Belgium; 3 A-Sense Lab, University of Antwerp, Groenenborgerlaan 171, 2020 Antwerp, Belgium; 4 Nanolab Centre of Excellence, University of Antwerp, Groenenborgerlaan 171, 2020 Antwerp, Belgium; 5 Astbury Centre for Structural Molecular Biology, University of Leeds, Leeds, LS2 9JT UK; 6 School of Molecular and Cellular Biology, University of Leeds, Leeds, LS2 9JT UK

**Keywords:** DNA, Solution-state NMR, Mass spectrometry, Biophysical chemistry, Nucleic acids, Small molecules

## Abstract

Artemisinin (ART) is a vital medicinal compound that is used alone or as part of a combination therapy against malaria. ART is thought to function by attaching to heme covalently and alkylating a range of proteins. Using a combination of biophysical methods, we demonstrate that ART is bound by three-way junction and duplex containing DNA molecules. Binding of ART by DNA is first shown for the cocaine-binding DNA aptamer and extensively studied using this DNA molecule. Isothermal titration calorimetry methods show that the binding of ART is both entropically and enthalpically driven at physiological NaCl concentration. Native mass spectrometry methods confirm DNA binding and show that a non-covalent complex is formed. Nuclear magnetic resonance spectroscopy shows that ART binds at the three-way junction of the cocaine-binding aptamer, and that binding results in the folding of the structure-switching variant of this aptamer. This structure-switching ability was exploited using the photochrome aptamer switch assay to demonstrate that ART can be detected using this biosensing assay. This study is the first to demonstrate the DNA binding ability of ART and should lay the foundation for further work to study implications of DNA binding for the antimalarial activity of ART.

## Introduction

**Figure 1:**
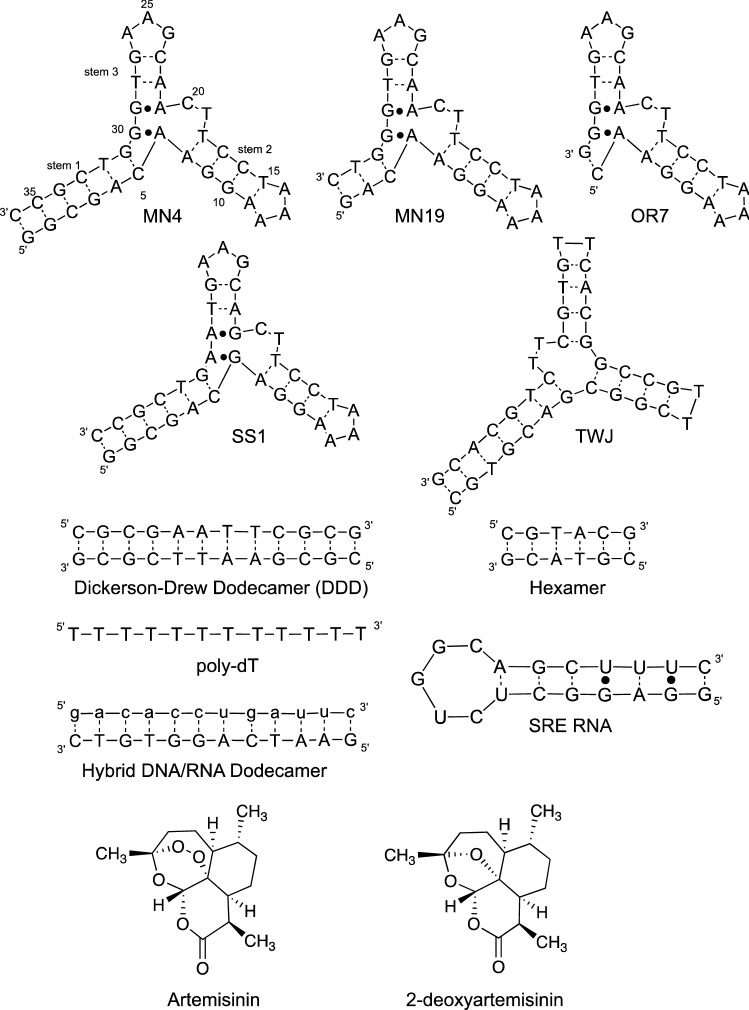
Secondary structures of the nucleic acid constructs used in this study and chemical structures of ART and 2-deoxyartemisinin. Dashes between nucleotides indicate Watson–Crick base pairs and dots indicate non-Watson–Crick base pairs. For the hybrid DNA/RNA dodecamer the RNA strand is shown in lowercase letters.

Artemisinin (ART) is derived from the plant *Artemisia annua* and is an important antimalarial compound used alone or with another antimalarial agent as part of artemisinin-based combination therapy (Fig. 1). Despite the importance of ART to world health, its mode of action is not fully known. It is thought that ART is a pro-drug and the endoperoxide bridge is cleaved through an iron-dependant mechanism inside the malarial parasite, possibly involving heme, resulting in alkylation of several different proteins^1^. This range of alkylated proteins is thought to be a reason why ART has a wide range of medicinal applications being studied such as efficacy against schistosomiasis, inflammation and as an anti-cancer agent. It is possible, and there has been speculation, that ART may alkylate DNA in *Plasmodia*, but this has not been demonstrated. DNA damage induced by the ART derivative artesunate has been identified as a possible contributor to its therapeutic effect against cancer cells^2^.

Aptamers are nucleic acid molecules that bind a ligand. They occur naturally as riboswitches^3^ or can be selected in the laboratory against virtually any target using the process called SELEX^4^. The cocaine-binding aptamer, distinct from most other aptamers, has a promiscuous ligand binding ability. This aptamer was selected to bind cocaine, and not cocaine metabolites^5,6^, yet binds quinine 50-fold tighter than cocaine^7,8^. This aptamer also tightly binds a range of other quinine-based ligands including amodiaquine, chloroquine and mefloquine^9^. Additionally, this aptamer binds levamisole weakly^10^ and also binds a fluorescent light-up compound (FPhOBtz) that specifically binds DNA three-way junctions^11^. The MN4 construct of this aptamer (Fig. 1) binds FPhOBtz tighter than any other DNA three-way junction analysed to date. The cocaine binding aptamer forms a three-way junction structure with a dinucleotide bulge and a tandem AG mismatch at the junction (MN4; Fig. 1). Another unusual feature of the cocaine-binding aptamer is that under conditions of zero to low NaCl concentration, the aptamer binds two copies of cocaine and quinine. This two-site binding occurs in an independent fashion, with the second, lower affinity, site involving stem 2^12^. The higher affinity binding site is at, or adjacent to, the three-way junction.

Another distinctive feature of the cocaine-binding aptamer is that its binding mechanism changes as the length of stem 1 varies. When stem 1 contains four base pairs or is longer, as in MN4 (Fig. 1), the aptamer is folded when it is not bound to its ligand. When stem 1 contains three or fewer base pairs (MN19 and OR7; Fig. 1) the aptamer is unstructured or dynamic, and ligand binding folds the aptamer in a ligand-induced, or structure-switching binding mechanism^13–15^. It is the short stem 1 version of the cocaine-binding aptamer that is most used in the many different biosensors constructed with this aptamer^16–20^.

In the work presented here, we show that ART is bound by the cocaine-binding aptamer as well as other DNA structures such as a generic three-way junction molecule and duplex DNA. ART does not bind single-stranded unstructured DNA (poly-dT) or either an RNA hairpin structure or a mixed DNA/RNA hybrid dodecamer (Fig. 1). We had initially looked at ART as a possible negative control for the binding observed by quinine derivatives, but surprisingly observed binding. Though binding was initially observed using ITC methods, it was also confirmed by NMR spectroscopy, UV-monitored thermal denaturation analysis and native mass spectrometry measurements as presented here. To the best of our knowledge this study is the first to demonstrate DNA binding by ART.

## Results and discussion

### Binding of artemisinin by the cocaine binding aptamer

**Figure 2:**
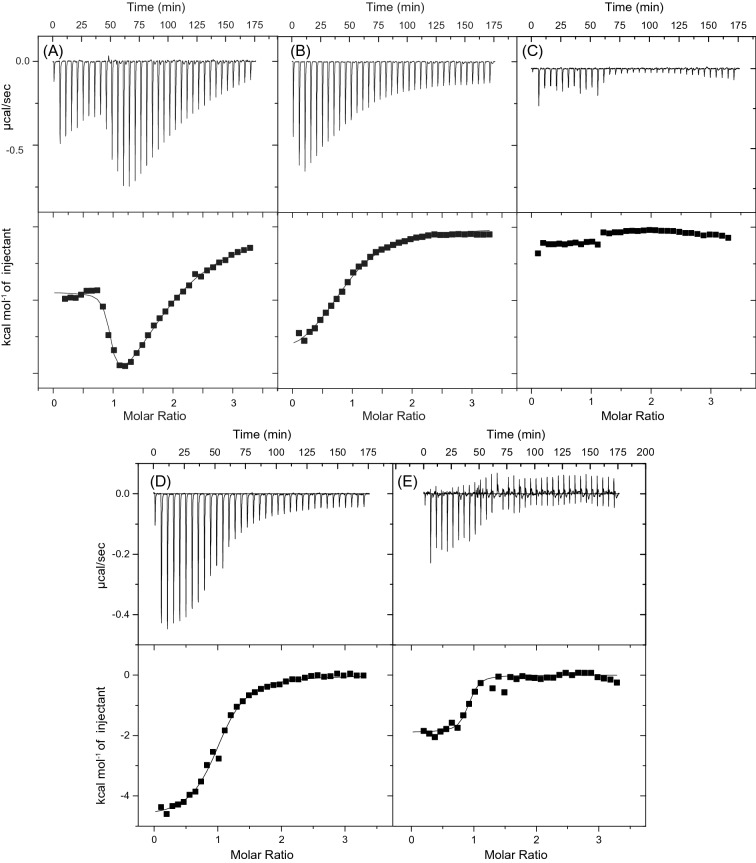
ITC thermograms showing interaction of ART with the MN4 aptamer in buffer containing 20 mM TRIS (pH 7.4), 5 mM KCl, 2.4% (v/v) DMSO, in (**A**) 140 mM NaCl and (**B**) 0 mM NaCl. (**C**) Interaction of 2-deoxyartemisinin with the MN4 aptamer in 20 mM TRIS (pH 7.4), 140 mM NaCl, 5 mM KCl, 1.5% (v/v) DMSO. (**D**) Quinine titrated into the MN4·ART complex. (**E**) ART titrated into the MN4·quinine complex. Unless otherwise specified, ITC competition experiments were acquired in buffer containing 20 mM TRIS (pH 7.4), 140 mM NaCl, 5 mM KCl, 1% DMSO. All ITC data were acquired at 15 °C.

ITC experiments were performed to determine if ART binds to the MN4 cocaine-binding aptamer. Based on the non-sigmoidal shape of the thermogram (Fig. 2A) we see that binding occurs and that two molecules of ART are bound by the aptamer. Additionally, from the shape of the curve “dipping down”, we can conclude that the weak binding site is more exothermic. Two alternative models were attempted to fit the data: (1) a two-site cooperative model and (2) a two independent sites binding model. Only the independent two-site model was able to fit the data. The resulting thermodynamic parameters from the fits are presented in Table 1. The binding affinity of ART to MN4 is 3.7-fold tighter than that for quinine and about 180-fold tighter than cocaine. It is roughly the same affinity as for chloroquine and mefloquine and weaker than the affinity of 7 nM that MN4 has for amodiaquine^9^. Binding thermodynamics indicate that ART binds to the MN4 aptamer with both a favourable entropy and enthalpy, a trait only shared with chloroquine, amodiaquine and primaquine amongst the ligands we have studied by ITC^9^. The second binding site in MN4 for ART is more exothermic than the first site, and it has an unfavorable entropy of binding, resulting in it being weaker (Table 1).

**Table 1:** Comparison of binding affinities and thermodynamic properties of ART binding by different nucleic acid structures used in this study.

Sample	K_d1_ (µM)	ΔH_1_ (kcal mol^−1^)	− TΔS_1_ (kcal mol^−1^)	K_d2_ (µM)	ΔH_2_ (kcal mol^−1^)	− TΔS_2_ (kcal mol^−1^)
MN4	0.03 ± 0.01	− 4.4 ± 0.2	− 5 ± 1	13 ± 4	− 15 ± 1	9 ± 1
MN19	0.02 ± 0.01	− 0.9 ± 0.1	− 9 ± 1	5 ± 1	− 11 ± 1	4 ± 1
OR7	0.03 ± 0.01	0.4 ± 0.1	− 9 ± 1	5 ± 1	− 22 ± 1	16 ± 1
SS1	0.004 ± 0.002	0.5 ± 0.1	− 12 ± 1	2 ± 1	− 7 ± 1	− 0.8 ± 0.4
TWJ	0.06 ± 0.02	− 0.1 ± 0.1	− 9 ± 1	10 ± 3	− 14 ± 6	7.4 ± 6
DDD	0.10 ± 0.03	− 0.22 ± 0.03	− 9 ± 1	31 ± 8	− 12 ± 4	6 ± 4
Hexamer	0.007 ± 0.004	0.7 ± 0.1	− 11 ± 1	3 ± 1	− 6 ± 1	− 2 ± 1
poly-dT	No binding observed					
SRE RNA	No binding observed					
Hybrid DNA/RNA	No binding observed					

Data acquired at 15 °C in 20 mM TRIS (pH 7.4), 140 mM NaCl, 5 mM KCl, 2.4% (v/v) DMSO.

### Dependence of binding affinity with NaCl concentration

Previously, we showed that decreasing the NaCl concentration in buffer increases the binding affinity of quinine, cocaine and primaquine while decreases affinity for amodiaquine, chloroquine and mefloquine^9,12^. ITC was used to investigate how ART interacts with the MN4 aptamer in a buffer with no NaCl present. In this buffer, the affinity of ART for the aptamer is decreased ~ 100-fold to (3.7 ± 0.3) µM compared with having 140 mM NaCl present, and only one molecule of ART is bound (Fig. 2B). In these conditions, the binding enthalpy becomes more favorable ((− 9.5 ± 0.3) kcal mol^−1^), while the binding entropy becomes unfavorable, − TΔS = (2.3 ± 0.3) kcal mol^−1^. This change is consistent with the behaviour of amodiaquine, chloroquine and mefloquine, whose binding affinity becomes weaker, binding enthalpy is more favorable and binding entropy is less favorable with decreasing NaCl concentration^9^. However, this behaviour is opposite from that seen for cocaine and quinine, where decreasing the NaCl concentration results in the ligand affinity increasing and the second binding site becoming observable^12^. This decrease in affinity as the NaCl concentration is decreased, as well as having a favourable binding entropy at 140 mM NaCl, indicates that ART binding by MN4 is hydrophobically driven.

### Importance of the endoperoxide bridge in ART binding

The importance of the endoperoxide bridge to the binding of ART by the MN4 DNA aptamer was assessed by testing the binding of 2-deoxyartemisinin (Fig. 1) by ITC. The thermogram of 2-deoxyartemisinin titrated into MN4 (Fig. 2C) does not show binding occurring. 2-deoxyartemisinin does not function as an antimalarial agent^21,22^ and these results demonstrate that the presence of the endoperoxide bridge is essential for binding of ART by the MN4 aptamer.

### Competition binding experiments

To determine whether quinine and ART share the same binding site in the cocaine-binding aptamer, we performed competition binding experiments using ITC. In the first experiment, ART was bound to MN4 in a 3:1 molar ratio, and quinine was then titrated in excess (Fig. 2D). We observe single-site binding that indicates the presence of bound ART does not prevent quinine binding. Using a competitive binding model, we show that quinine binds to the cocaine-binding aptamer with an apparent dissociation constant ( K_d, app_) of (1.1 ± 0.1) µM. In the second experiment, quinine was pre-bound to MN4 in a 3:1 molar ratio, and ART titrated to excess. In this case, a sigmoidal curve is also observed indicating that one molecule of ART binds to the quinine-bound MN4 aptamer with a K_d, app_ of (0.5 ± 0.2) µM (Fig. 2E). These data are interesting in that they show the quinine and ART are not completely competitive with each other in binding MN4. Our previous competition experiments with quinine displacing cocaine^8^ and amodiaquine displacing quinine^9^ did show competitive binding indicating these ligands share a common binding site, at 140 mM NaCl concentration. In contrast, the binding sites of quinine and ART do not completely overlap since quinine can bind despite the presence of the higher affinity ART bound to MN4. However, the presence of quinine does prevent one molecule of ART binding.

### A non-covalent complex is formed

**Figure 3:**
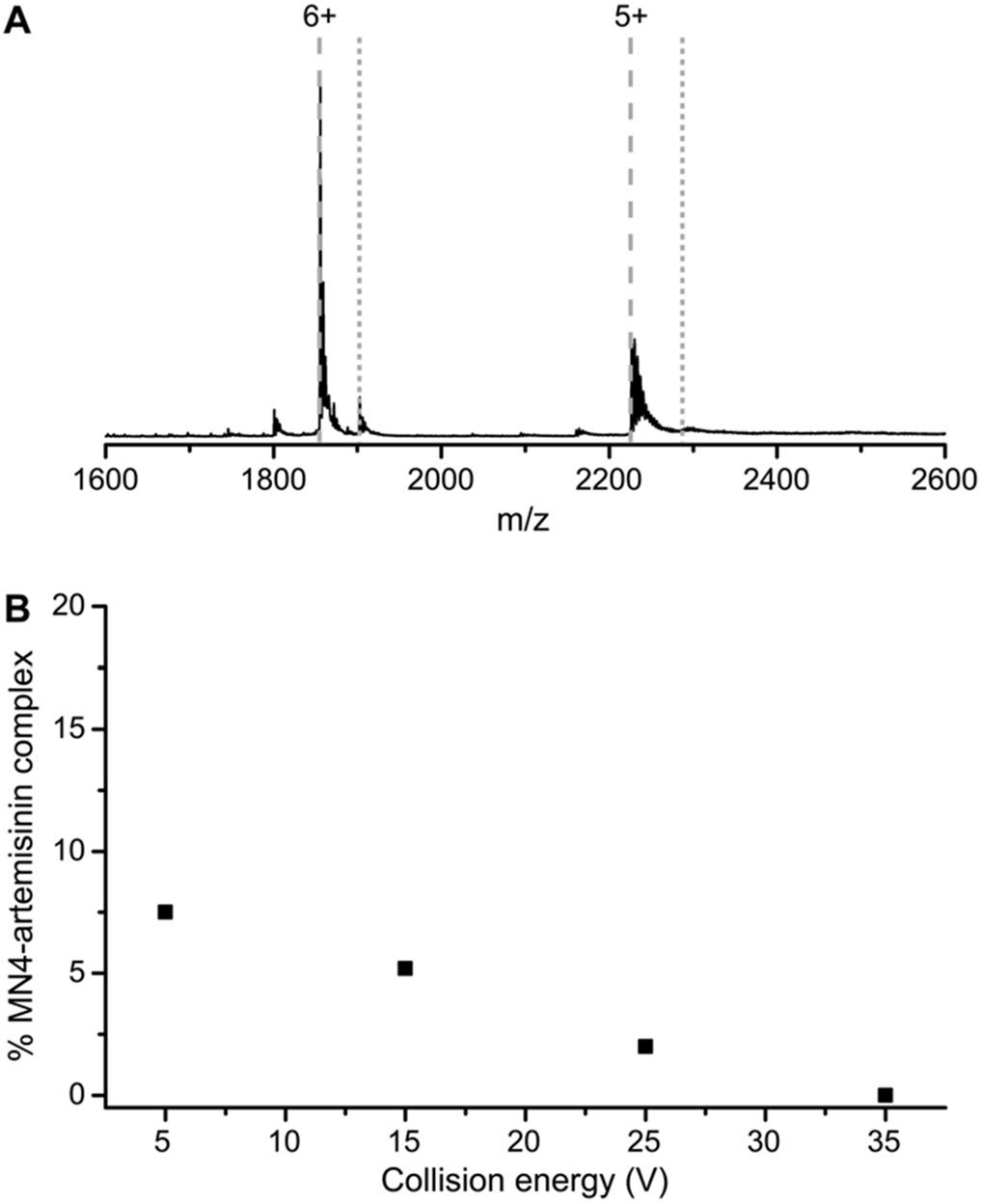
Native mass spectrometry showing the interaction between ART and the MN4 DNA aptamer forms a non-covalent complex. (**A**) Native mass spectrum of the MN4 aptamer with ART (1:10 aptamer:ART ratio) in 300 mM ammonium acetate. Theoretical m/z-values of the apo form (dashed lines) and the 1:1 stoichiometry of the complex (dotted lines) are indicated for the 6+ and 5+ charge states. (**B**) The percentage of observed MN4·ART complex in relation to the total aptamer signal, i.e., unbound and 1:1 bound peaks, plotted in function of the trap collision energy (i.e. acceleration voltage).

Native mass spectrometry experiments were performed to verify whether ART is bound by the MN4 aptamer and to determine if this binding is covalent or non-covalent. Figure 3A shows the mass spectrum of the aptamer after addition of the target at 10 times molar excess. The aptamer is detected with charge states 6 + (*m/z* = 1855.7) and 5 + (*m/z* = 2226.7) and the complex with a 1:1 binding stoichiometry is detected at *m/z* = 1909.8 and *m/z* = 2291.5 for the 6+ and 5+ charge state, respectively. Previously, the binding of quinine to a set of cocaine-binding aptamers was observed using mass spectrometry techniques^23^. Here, the interaction of ART by MN4 is also detected. This also demonstrates that nucleic acid-small molecule complexes that are predominately driven by hydrophobic interactions, as in the ART-MN4 complex, can be detected by native mass spectrometry techniques as well as enthalpically driven interaction as seen for the quinine-MN4 complex.

To verify whether ART is covalently bound to the aptamer, collision-induced dissociation (CID) experiments were performed. In CID, the acceleration voltage applied to the analyte, the collision energy, is increased causing disruption of the weakest (noncovalent) bonds. When CID is applied to complexes, the noncovalent interactions holding the complex together are broken and the complex dissociates^24^. Supplemental Fig. S1 shows the mass spectra recorded at increasing collision energies. Based on these spectra, Fig. 3B shows the amount of complex present as a function of the collision energy. We observe that when raising the energy, the amount of complex decreases from 15 V onwards. This means that the complex dissociates, until no complex remains at 35 V. At these low energies, only noncovalent interactions are disrupted indicating that ART is non-covalently bound by the MN4 aptamer. If covalent binding occurred, other backbone fragments would appear in the mass spectrum together with the loss of ligand, which is not observed.

### Binding of artemisinin to different DNA structures

Given the initially surprising finding that ART is bound by the MN4 aptamer we tested the binding of ART by other DNA structures to explore whether ART is specific for the MN4 cocaine-binding aptamer or if it interacts with other DNA structures. We looked at other three-way junction structures, duplex DNA, single stranded DNA, an RNA hairpin structure, and a hybrid DNA/RNA duplex. (Fig. 1). As examples of three-way junctions, we chose two cocaine-binding aptamer variants with different stem 1 lengths (MN19 and OR7), a slightly modified version of the cocaine-binding aptamer previously used as a negative control for cocaine and quinine binding (SS1) and generic three-way junction structure (TWJ) (Fig. 1). ITC experiments show that two molecules of ART are bound by the cocaine-binding aptamer regardless of the length of stem 1 as evident in non-sigmoidal binding curves for MN19 and OR7 (Supplemental Fig. S2, Table 1). We also note the similar shape of these thermograms to that for MN4 (Fig. 2A) where the weaker binding site is more exothermic than the tighter binding site. These results demonstrate that ART can bind these variants (MN19, OR7) of the cocaine-binding aptamer, though do not yet indicate that these constructs undergo ligand-induced folding.

ITC methods also demonstrate that the SS1 and TWJ DNA structures bind ART (Supplemental Fig. S3, Table 1). Binding of ART by the SS1 construct is significant as SS1 has been used as a negative control for the cocaine-binding aptamer since cocaine and quine both bind very weakly, with the K_d_ value being in the hundreds of micromolar range^25^. Binding of ART by SS1 is consistent with quinine and ART not being competitive with each other and ART interacting with the MN4 structure in a different manner than cocaine and the quinine-based ligands. The generality of ART binding by DNA three-way junction constructs is shown by the construct TWJ also binding ART (Supplemental Fig. S3, Table 1). TWJ has been used previously as a model system for DNA three-way junctions^26^.

The binding of ART to DNA structures was further explored using two duplex DNA molecules, the self-complementary Drew-Dickerson dodecamer (DDD)^27^ and a self-complementary hexamer (Fig. 1). ITC experiments show that two molecules of ART bind to both duplexes (Supplemental Fig. S3, Table 1). These binding experiments show that ART binds tighter to the hexamer than the dodecamer (Table 1).

This study was further extended to see if the poly-dT molecule, a model for unstructured single-stranded DNA and the smaug recognition element (SRE) RNA stem loop molecules bind ART individually. As shown by ITC, neither of these molecules bind ART (Supplemental Fig. S3, Table 1). These results demonstrate that ART is bound by duplex DNA-containing molecules, but not single-stranded DNA or by an RNA stem loop molecule. As the RNA stem loop forms an A-form helix^28^ we tested whether ART binds a hybrid DNA/RNA duplex (Fig. 1) that also forms an A-form helix^29^. As seen in Supplemental Fig. S3 ART shows no interaction with this hybrid DNA/RNA dodecamer. Together, these data show that ART binds B-form duplex molecules but not nucleic acids in A-form helical geometry.

### ART binds at the three-way junction in the cocaine-binding aptamer

**Figure 4:**
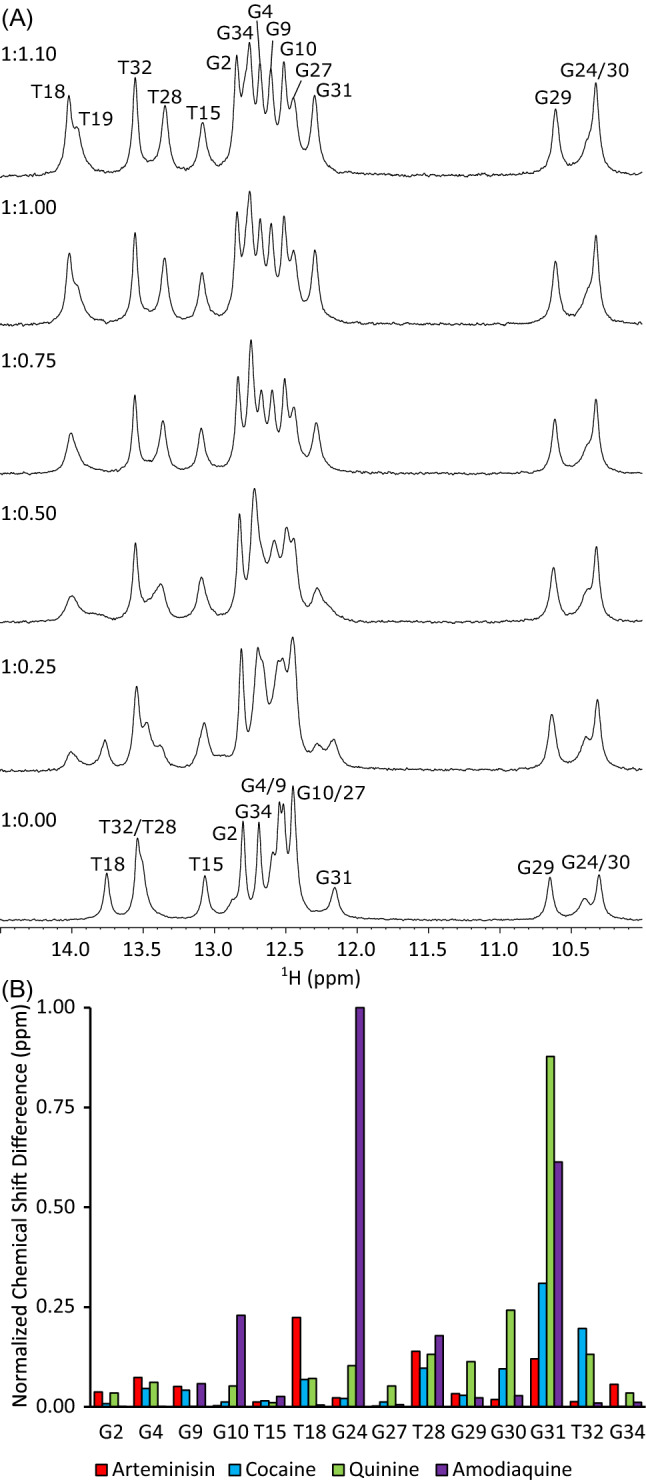
(**A**) ^1^H NMR spectra showing the imino proton resonances of MN4 as ART is titrated into the sample up to a 1:1.1 molar ratio of aptamer to ligand. Sample contained 1.4 mM aptamer, 20 mM H_x_Na_y_PO_4_, pH 7.4, 10% D_2_O. Final titration point contains ~ 3% DMSO-d_6_. Spectra acquired at 5 °C. (**B**) Histograms showing the absolute value of the difference in peak position of the imino protons for free and ligand-bound MN4. Data is normalized to the largest chemical shift difference value. Displayed ligands are ART (red), quinine (green), cocaine (blue) and amodiaquine (purple). The cocaine, amodiaquine and quinine change in chemical shift data are based on previous published data^9,14^.

To gain structural insight into how the MN4 cocaine-binding aptamer interacts with ART we analysed the NMR spectroscopy chemical shift perturbations from a titration of MN4 with ART (Fig. 4A). In the free MN4, the imino region of the MN4 aptamer looks virtually identical to what we observed and assigned previously^14,15,30^. As ART is titrated into MN4 to a final molar ratio of 1:1.1 aptamer:ligand (Fig. 4A), some imino resonances changed chemical shift. For example, G31 shifts downfield. During the titration, the G31 imino proton is in slow exchange on the NMR timescale between the free and bound forms. This finding is consistent with the tight binding of ART by MN4. The imino proton resonances of the 1:1.1 molar ratio MN4:ART complex were assigned using a 2D ^1^H–^1^H NOESY spectrum (Supplemental Fig. S4).

A histogram of the movement of the imino proton resonances between the free MN4 and ligand-bound MN4 was constructed to compare ART binding with that of previously studied ligands (Fig. 4B). This was done for ART using the data in this study, and for cocaine, quinine and amodiaquine using previously published data^9,14,15,30^. For ART, the resonances that shifted the most with binding are T18, T28, and G31. Those resonances are from nucleotides close to the three-way junction. Both the quinine-, cocaine- and amodiaquine-bound forms of MN4 also have resonances around the three-way junction that move most with ligand binding though the specific resonances and the value of the chemical shift they move differs between ligands (Fig. 4B).

### ART triggers ligand-induced folding in the MN19 cocaine-binding aptamer

To confirm that MN19 undergoes ligand-induced folding with ART binding we monitored the imino region of its 1D ^1^H NMR spectrum as ART was titrated to a molar ratio of 1:2 (aptamer:ART) (Supplemental Fig. S5). As seen previously for MN19-binding other ligands, the binding of ART tightened up the “loose” structure of MN19, as observed by the number of peaks in the spectrum of the free MN19 aptamer increasing as ligand is added. Specifically, the imino proton resonances of G9, G10, T17, T18, T19, T28, G29, G30, and G31 all appear as ART is added (Supplemental Fig. S5). Additionally, the intensity of peaks present in the free MN19 spectrum (G24, G27, T28, and T32) increase in intensity as ART is added.

The thermal stability of the ART-bound MN19 was gauged by recording the 1D ^1^H-NMR spectrum with increasing temperature. The imino proton resonances were monitored as the temperature was increased from 3 to 35 °C (Supplemental Fig. S6). Imino proton resonances start to broaden out at 9 °C with T15 and G31 disappearing first. At 15 °C, T18, T19, and T28 disappear and G9, G10, G24, G29, and G30 have also broadened out significantly. At 20 °C, only G27 and T32 can be observed with G27 broadening out shortly afterwards. G27 is visible up to 30 °C where it broadens out quite significantly, and then disappears by 35 °C. The resonances in ART-bound MN19 disappear approximately 5 °C lower than in quinine-bound MN19 indicating the complex with ART is slightly less thermally stable^14^.

### ART binding stabilizes the structure of the MN19 cocaine-binding aptamer

**Figure 5:**
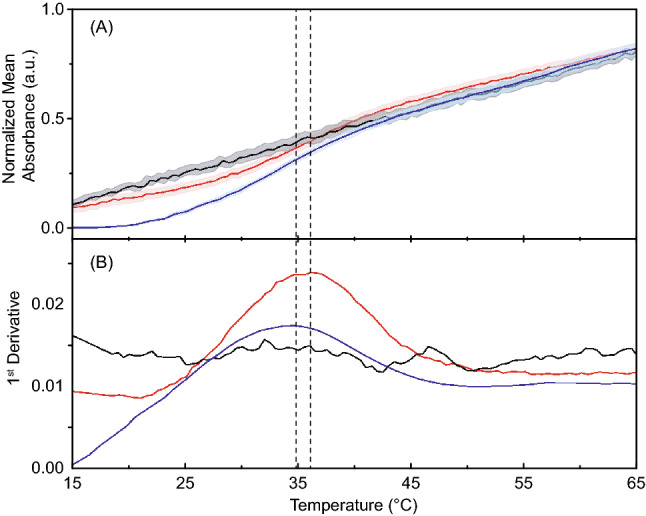
Analysis of the thermal stability of MN19 free and ligand-bound using UV melting plots. Shown in (**A**) are the normalized average of UV absorbance values at 260 nm versus temperature for the unbound MN19 (black), ART-bound MN19 (blue), and quinine-bound MN19 (red) as a positive control. Displayed in (**B**) are the first derivative plots. Dashed lines indicate the obtained T_m_ points of the aptamer. Data acquired in PBS (pH 7.4), 3% (v/v) acetonitrile. Each data point denotes an average of three experiments with the error ribbons corresponding to one standard deviation.

The binding of ART by MN19 and stabilisation of MN19 cocaine-binding aptamer was demonstrated by measuring the thermal stability of the unbound MN19 as well as the ART-bound and quinine-bound MN19 aptamers (Fig. 5). The DNA UV absorbance at 260 nm of unbound MN19 aptamer does not show a shift observed previously^31^, and as expected for a loosely folded or unfolded nucleic acid molecule. For free MN19, the UV absorption increases linearly with temperature (Fig. 5). The MN19·ART complex shows a sigmoidal transition from folded to unfolded molecule with a thermal denaturation point at (34.8 ± 1.1) °C. The MN19·quinine complex was tested as the positive control, where it shows a sigmoidal transition with a thermal denaturation at (36.4 ± 1.2) °C. This unfolding temperature of MN19·quinine is consistent with our prior measurements^13,31^. These data confirm ART binding by the MN19 cocaine-binding aptamer and confirm the ART complex is slightly less thermally stable than with quinine as suggested by the NMR-monitored melt.

### Detection of ART using the photochrome aptamer switch assay

**Figure 6:**
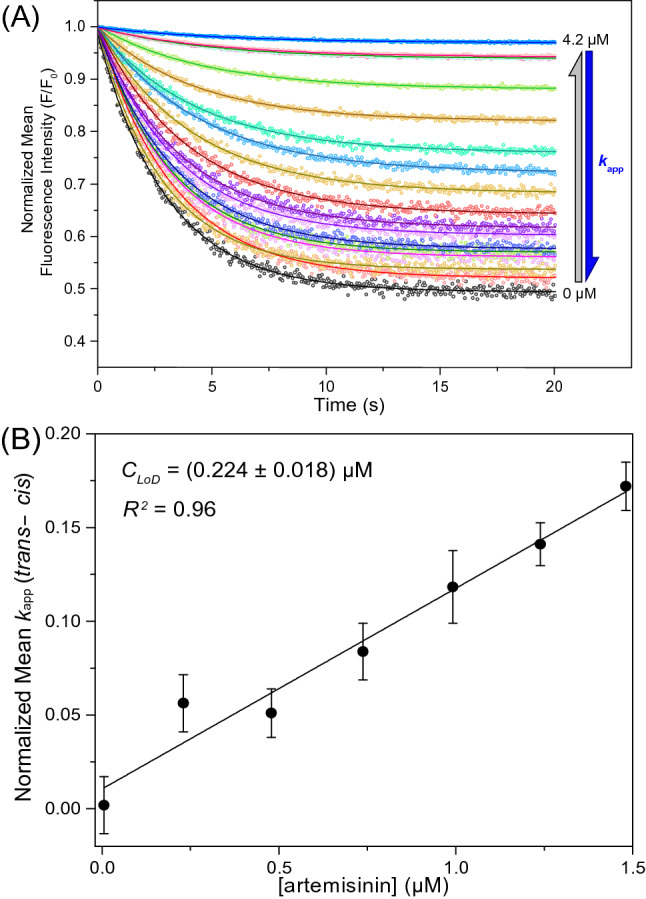
Detection of ART using the photochrome aptamer switch assay. Panel (**A**) displays fluorescence decay plots of 0.1 µM unbound MN19-SITS (black) and as a function of zero to 4.2 µM ART concentration (light blue). The MN19-SITS is continuously excited at 340 nm and the emission at 422 nm are simultaneously detected as a function of time. Each normalized decay plot is fitted to the first-order decay function (solid lines) to quantify the apparent *trans*–*cis* decay kinetics (Eq. 1; *k*_app_). Panel (**B**) calibration plot for the normalized average *k*_app_ values of MN19-SITS against ART concentrations. The concentration limit of detection (C_LoD_) obtained is (0.22 ± 0.02) µM. Triplicated experiments were performed in 20 mM Tris (pH 7.4), 140 mM NaCl at 20 °C. The error bars correspond to one standard deviation.

As the NMR and UV melt data indicate the folding of MN19 with ART binding, we applied the photochrome aptamer switch assay (PHASA)^32^ for MN19-SITS both free and ART-bound in PBS plus 3% (v/v) DMSO at 20 °C and constant instrumental settings (λ_*ex*_ = 340 nm and λ_*em*_ = 422 nm). We measured the fluorescence decay of MN19-SITS to detect the kinetics rate due to internal conversion of *trans*- to *cis*-stilbene. First-order decay analysis was used to determine the apparent kinetics rates (*k*_*app*_) (Fig. 6A). The ratio of average *k*_*app*_, in the presence of ART, to average *k*_*app*_ of unbound MN19-SITS were analyzed as a function of ART concentration (Supplemental Fig. S7). The observed linear trend shows that the apparent rate for MN19-SITS decreases as the ART concentration increases. This observation is consistent with the presence of ART inducing the folding of MN19-SITS at 20 °C. Furthermore, we obtained a linear slope of ((− 1.6 ± 0.13) × 10^−1^) µM^−1^ (Supplemental Fig. S7). The quantified slope agrees with previously published data for MN19-SITS bound to cocaine^32^.

We analyzed the concentration limit of detection (*C*_*LoD*_) for ART in buffer utilizing the PHASA method with MN19-SITS (Fig. 6B). Using the linear portion of the dose–response calibration plot for the mean normalized *k*_*app*_ versus ART concentration, *C*_*LoD*_ is determined as (0.22 ± 0.02) µM, which is 38-fold more sensitive than previously reported *C*_*LoD*_ for MN19-SITS binding cocaine. We attribute this increase in the detection sensitivity to the fact that MN19 binds ART tighter than it does with cocaine^32^. Together these data show that ART binding by MN19 can be exploited in a sensing application for the detection of ART.

## Conclusions

In this study, we show that the antimalarial compound ART binds DNA molecules that contain duplex DNA structures. We initially saw binding with the cocaine-binding aptamer using a combination of ITC, NMR spectroscopy, UV melt, fluorometry and native mass spectrometry methods. Though the endoperoxide bridge is required for binding, indicating that ART binding might result in a covalently bound ligand in a similar fashion as ART has been proposed to covalently bind heme, collision-induced dissociation mass spectrometry experiments did not indicate the presence of covalent binding.

We then showed the binding of ART is not specific to the cocaine-binding aptamer, but it is a general property of molecules containing duplex DNA. We demonstrate that various three-way junction molecules all bind ART as well as a duplex forming dodecamer and a hexamer. Only single stranded DNA (poly-dT) an RNA hairpin molecule and a hybrid DNA/RNA duplex did not bind ART. This non-specific biding is consistent with the NMR results showing binding of ART near the three-way junction of MN4 but not at the same location as quinine or cocaine. This is also consistent with the ITC competition experiments showing that quinine and ART are not competitive with each other.

Despite ART not being specific for the cocaine binding aptamer, the binding of ART by MN19 still results in ligand induced-folding in this aptamer. This occurs as ART binds at the three-way junction and likely pulls together or stabilizes the stems in the molecule. This MN19 folding with ART binding can be exploited by the PHASA assay previously used to quantify cocaine binding and indicates other assays that rely on short stem 1 constructs of the cocaine-binding aptamer folding will likely also detect ART.

The implication of ART binding these DNA molecules for the role of ART as an antimalarial agent is yet to be revealed and can be the focus of future work. Whatever role DNA binding by ART may have, depends on numerous factors such as availability and pharmacology of ART in different biological systems.

## Methods

### Materials

All DNA samples for analysis (except MN4 for native mass spectrometry analysis, see below) were purchased from Integrated DNA Technologies (IDT, Coralville, Iowa) with standard desalting. The identity of aptamer samples was confirmed by mass spectrometry by the manufacturer. For each 1 micromole scale synthesis, the DNA is dissolved in ~ 1 mL of distilled deionized H_2_O (ddH_2_O) and then exchanged 3 times against 1 M NaCl using 3 kDa molecular weight cut off Amicon-style concentrators to compete off any unwanted substances bound to the nucleic acid. The sample is then exchanged 4–6 times against ddH_2_O. Aptamer samples were finally exchanged 3–4 times against buffer (20 mM TRIS (pH 7.4), 140 mM NaCl, 5 mM KCl). The aptamer concentration was measured using UV spectroscopy and the known extinction coefficient provided by the manufacturer. The SRE-RNA hairpin was produced by T7 RNA polymerase from a DNA template in a large-scale in vitro transcription reaction using methods described previously^28,33^.

Small molecule ligands and the SITS (4-acetamido-4′-isothiocyanato-2,2′-stilbenedisulfonic acid disodium salt) used in this study were obtained from Sigma Aldrich. Stock solutions of ART and 2-deoxyartemisinin were prepared in 100% DMSO. All the SITS powder and solutions were kept under dark conditions to prevent random photoisomerization of *trans*-SITS. To covalently attach SITS to the MN19 aptamer in the photochrome aptamer switch assay (PHASA) experiments, the MN19 DNA was synthesized with a C_6_-NH_2_ group at the 5′ end.

### Isothermal titration calorimetry

ITC binding experiments were performed using a MicroCal VP-ITC instrument in a manner similar to what we have previously described^34^. Samples were degassed before analysis with a MicroCal Thermo Vac unit for 5 min to avoid large spikes in the ITC baseline due to air bubble formation during the experiment. All experiments were corrected for the heat of dilution of the titrant. Titrations were performed with aptamer samples in the cell and the ligand, as the titrant, in the needle. All aptamer samples were heated in a 95 °C water bath for 3–5 min and cooled in an ice water bath for at least 10 min prior to use in a binding experiment to allow the DNA aptamer to anneal in an intramolecular fashion. The binding experiments were performed at 15 °C with the aptamer solution at 20 µM using a ligand concentration of 0.312 mM. Both ART and 2-deoxyartemisinin were diluted to the experimental concentration with the same buffer (20 mM TRIS (pH 7.4), 140 mM NaCl, 5 mM KCl) to a final DMSO concentration of 1–2.4% (v/v). DMSO was added to the aptamer solution and the reference cell at the same concentration to avoid buffer mismatch. All binding experiments consisted of an initial delay of 60 s, first injection of 2 µL and 300 s delay. The subsequent 34 injections were of 8 µL, spaced every 300 s. The first point was removed from all data sets due to the different injection volume and delay parameters. ITC data following a one-site binding model were fit using Origin 7 software provided by the manufacturer. Two-site binding data were fit to a two-site independent binding model developed by Freiburger et al. using Matlab 14 software^35^.

### NMR spectroscopy

NMR experiments were conducted on a 600 MHz Bruker Avance spectrophotometer in a manner previously described^36^. Prior to performing NMR experiments, samples were heated in a 90 °C water bath for 3 min then cooled in an ice-water bath to favour intramolecular aptamer folding. Spectra were acquired in 20 mM H_x_Na_y_HPO_4_, pH 7.4 in 10% ^2^H_2_O, 90% H_2_O. NMR data were processed using TopSpin 3.7 (Bruker).

A 1.4 mM sample of MN4 was titrated with ART to an aptamer:ligand molar ratio of 1:1.1. A ^1^H–^1^H NOESY (200 ms mixing time) was performed on the ART-bound MN4 sample to assign the imino proton resonances. Next, the ART-bound MN4 sample was titrated with additional ART up to a final molar ratio of 1:2 (aptamer:ligand). Finally, the ART-bound MN4 sample was titrated with sodium chloride up to a concentration of 140 mM sodium chloride.

A 400 μM sample of MN19 was titrated with ART to a molar ratio of 1:2 (aptamer:ligand). Next, the base pair stability of the ART bound MN19 aptamer was monitored by increasing the temperature of the sample. The first scan was acquired at 3 °C and the temperature was raised in 3° increments to 15 °C, then in 5 °C increments to 35 °C when the imino proton resonance were no longer observed.

### Aptamer-SITS conjugation and fluorescence decay kinetics

The MN19-C_6_-NH_2_ aptamer was covalently conjugated to *trans*-SITS through the amine-isothiocyanate reaction as previously described^32^. The aptamer-SITS product was washed and concentrated 10 times using ultracentrifugation. To confirm the equimolar ratio of MN19 to SITS products, the relative UV absorbance at 260 nm and 340 nm of aptamer-SITS were measured. All solutions were filter-sterilized using a 0.2 μm microfilter.

The fluorescence decay kinetics of 0.1 µM MN19-SITS were analyzed in 20 mM Tris buffer (pH 7.4), 140 mM NaCl, 3% v/v DMSO at 20 °C as a function of zero to 4.2 µM ART concentration employing a Cary Eclipse fluorescence spectrophotometer and 10-mm fused quartz cuvettes. Since MN19 and ART had no light absorbance at (340 ± 10) nm, the inner-filter effect for the loss of the excitation light intensity was not considered. An unbound ART solution in the binding buffer was used as the blank. The experimental and blank samples were excited at 340 nm and emitted light intensities at 422 nm were simultaneously detected as a function of time in milliseconds. The obtained fluorescence decay kinetics were normalized as *F*/*F*_*o*_ and fitted to the first-order decay function:

1 $$F = F_{o} e^{{ - k_{app} t}}$$

where *F*_*o*_ is the fluorescence intensity at *t* = *0*, and *k*_*app*_ is the apparent fluorescence decay rate in s^−1^. The quantified average *k*_*app*_ values for each titration point were normalized and analysed against ART concentration. The concentration limit of detection (*C*_*LoD*_) of ART was quantified from the residual standard deviation of the regression data obtained from the linear region of the dose–response curve as:

2 $$C_{LoD} = 3S_{y/x} /m$$

where *S*_*y/x*_ is the standard deviation of the linear regression residuals, and *m* is the fitted linear slope. Additionally, the ratio of *k*_*app*_ in the presence of ART to *k*_*app*_ of unbound MN19-SITS were analyzed as a function of ART concentration and fitted to a linear plot. The obtained linear slope was used to compare with previously published values for ligand-induced folding of the MN19 aptamer binding cocaine^32^.

### UV-monitored thermal denaturation

UV melt experiments of the unbound and ART-bound MN19 aptamer, were performed using a Cary 100 UV–Vis spectrometer and 10-mm fused quartz cuvettes. Each experiment was performed in three trials in PBS buffer (10 mM sodium phosphate buffer, pH 7.4, 2.7 mM KCl, 137 mM NaCl). 3% v/v acetonitrile (ACN) added to the buffer instead of DMSO to avoid errors caused by the freezing point of DMSO at ~ 19 °C. All solutions were filter-sterilized using a 0.2 μm microfilter. The rate of temperature increased at 1 °C/min and controlled by a Cary Peltier unit. Two data points were acquired per minute in a temperature range from 15 to 75 °C. Data were analysed in a range of 15 °C to 65 °C to eliminate background signals associated with the boiling temperature of acetonitrile. For each trial, (1.7 ± 0.1) µM MN19 was chosen to yield ~ 0.5 absorbance arbitrary units (a.u.) at 260 nm using the extinction coefficients of the aptamer. The ligand to aptamer molar ratio was kept constant at 95% ligand-bound using Eq. (3):

3 $$X = \left[ {\text{L}} \right]^{n} /\left( {{\text{K}}_{{\text{d}}}^{n} + \left[ {\text{L}} \right]^{n} } \right)$$

where [L] is the ligand concentration, *X* is the fraction bound, *n* equals 2 binding events and K_d_ is the dissociation constant at 15 °C as determined in this study using ITC methods. Quinine was used as the positive control ligand, and ART in PBS plus 3% ACN was used as the blank for MN19·ART experiments. For the unbound MN19 experiments, PBS plus 3% ACN was used as the blank. To quantify the thermal denaturation points (T_m_), the obtained data from blanks were subtracted from their corresponding experimental values, and the first-order derivative of each thermal curve was plotted as a function of temperature using OriginPro 2016 software (OriginLab Corporation, Northhampton, MA, USA) as described previously^31^.

### Mass spectrometry

The MN4 DNA aptamer was purchased from Eurogentec (Belgium) purified with a differential precipitation purification method. Ammonium acetate solution (7.5 M, molecular biology grade), ART (98%) and quinine hydrochloride dihydrate (≥ 98.0%) were purchased from Sigma-Aldrich (Bornem, Belgium). All solutions were prepared using ddH_2_O. The aptamer was buffer exchanged into 300 mM ammonium acetate pH 6.8 using Micro Bio-spin columns (Bio-gel P6, Bio-Rad). The concentration of the aptamer was verified using a NanoPhotometer N60 (Implen). Extinction coefficients were calculated by the NanoPhotometer NPOS software (Implen) based on the oligonucleotide sequence. A 50 mM stock of ART was prepared in DMSO (Fisher Scientific) and further diluted into 300 mM ammonium acetate (pH 6.8). Quinine was solubilized in 300 mM ammonium acetate (pH 6.8). Aptamer-target complexes were prepared using a 1:10 aptamer-target molar ratio with a final aptamer concentration of 10 µM.

Native mass spectrometry analyses were performed on a Synapt G2 HDMS Q-TOF instrument (Waters, Manchester, UK). Approximately 2–4 µL of sample was introduced into the mass spectrometer, using nano-electrospray ionization with in-house made gold-coated borosilicate glass tapered-tip capillaries. The following instrument settings were used: spray capillary voltage 1.3–1.6 kV, source temperature 30 °C, sampling cone 20 V, extraction cone 1 V, trap collision energy 5–35 V, transfer collision energy 0 V, trap DC bias 40 V, IMS wave height 35 V and IMS wave velocity 800 m/s. The backing pressure was set to 2.79 mbar, the source pressure to 2.42 × 10^−3^ mbar, the trap pressure to 2.38 × 10^−2^ mbar, the IMS pressure to 3.00 mbar and the transfer pressure to 2.46 × 10^−2^ mbar. All data were analyzed using MassLynx 4.2.

## Supplementary Information

Supplementary Figures.

## References

[1] Zhou Y, Li W, Xiao Y (2016). Profiling of multiple targets of artemisinin activated by hemin in cancer cell proteome. ACS Chem. Biol..

[2] Li PCH (2008). Artesunate derived from traditional Chinese medicine induces DNA damage and repair. Can. Res..

[3] Breaker RR (2018). Riboswitches and translation control. Cold Spring Harb. Perspect. Biol..

[4] Li L (2021). Nucleic acid aptamers for molecular diagnostics and therapeutics: Advances and perspectives. Angew. Chem. Int. Ed..

[5] Stojanovic MN, de Prada P, Landry DW (2000). Fluorescent sensors based on aptamer self-assembly. J. Am. Chem. Soc..

[6] Slavkovic S, Altunisik M, Reinstein O, Johnson PE (2015). Structure-affinity relationship of the cocaine-binding aptamer with quinine derivatives. Bioorg. Med. Chem..

[7] Pei R (2009). High-resolution cross-reactive array for alkaloids. Chem. Commun..

[8] Reinstein O (2013). Quinine binding by the cocaine-binding aptamer. Thermodynamic and hydrodynamic analysis of high-affinity binding of an off-target ligand. Biochemistry.

[9] Slavkovic S, Churcher ZR, Johnson PE (2018). Nanomolar binding affinity of quinine-based antimalarial compounds by the cocaine-binding aptamer. Bioorg. Med. Chem..

[10] Shoara AA, Churcher ZR, Slavkovic S, Johnson PE (2021). Weak binding of levamisole by the cocaine-binding aptamer does not interfere with an aptamer-based detection assay. ACS Omega.

[11] Van Riesen AJ (2021). Visible fluorescent light-up probe for DNA three-way junctions provides host-guest biosensing applications. ACS Appl. Bio Mater..

[12] Neves MAD, Slavkovic S, Churcher ZR, Johnson PE (2017). Salt-mediated two-site ligand binding by the cocaine-binding aptamer. Nucleic Acids Res..

[13] Neves MAD (2017). Optimizing stem length to improve ligand selectivity in a structure-switching cocaine-binding aptamer. ACS Sens..

[14] Churcher ZR, Garaev D, Hunter HN, Johnson PE (2020). Reduction in dynamics of base pair opening upon ligand binding by the cocaine-binding aptamer. Biophys. J..

[15] Churcher ZR, Neves MAD, Hunter HN, Johnson PE (2017). Comparison of the free and ligand-bound imino hydrogen exchange rates for the cocaine-binding aptamer. J. Biomol. NMR.

[16] Stojanovic MN, de Prada P, Landry DW (2001). Aptamer-based folding fluorescent sensor for cocaine. J. Am. Chem. Soc..

[17] Baker BR (2006). An electronic, aptamer-based small-molecule sensor for the rapid, label-free detection of cocaine in adulterated samples and biological fluids. J. Am. Chem. Soc..

[18] Das J (2012). An ultrasensitive universal detector based on neutralizer displacement. Nat. Chem..

[19] Neves MAD, Blaszykowski C, Thompson M (2016). Utilizing a key aptamer structure-switching mechanism for the ultrahigh frequency detection of cocaine. Anal. Chem..

[20] Ahmadi Y, Soldo R, Rathammer K, Eibler L, Barišić I (2021). Analyzing criteria affecting the functionality of G-quadruplex-based DNA aptazymes as colorimetric biosensors and development of quinine-binding aptazymes. Anal. Chem..

[21] Robert A, Meunier B (1998). Is alkylation the main mechanism of action of the antimalarial drug artemisinin?. Chem. Soc. Rev..

[22] Balint GA (2001). Artemisinin and its derivatives: An important new class of antimalarial agents. Pharmacol. Ther..

[23] Daems E, Dewaele D, Barylyuk K, De Wael K, Sobott F (2021). Aptamer-ligand recognition studied by native ion mobility-mass spectrometry. Talanta.

[24] Benesch JLP, Aquilina JA, Ruotolo BT, Sobott F, Robinson CV (2006). Tandem mass spectrometry reveals the quaternary organization of macromolecular assemblies. Chem. Biol..

[25] Shoara AA, Slavkovic S, Donaldson LW, Johnson PE (2017). Analysis of the interaction between the cocaine-binding aptamer and its ligands using fluorescence spectroscopy. Can. J. Chem..

[26] Muhuri S, Mimura K, Miyoshi D, Sugimoto N (2009). Stabilization of three-way junctions of DNA under molecular crowding conditions. J. Am. Chem. Soc..

[27] Drew HR (1981). Structure of a B-DNA dodecamer: Conformation and dynamics. Proc. Natl. Acad. Sci. USA.

[28] Johnson PE, Donaldson LW (2006). RNA recognition by the Vts1p SAM domain. Nat. Struct. Mol. Biol..

[29] Davis RR, Shaban NM, Perrino FW, Hollis T (2015). Crystal structure of RNA-DNA duplex provides insight into conformational changes induced by RNase H binding. Cell Cycle.

[30] Neves MAD, Reinstein O, Johnson PE (2010). Defining a stem length-dependant binding mechanism for the cocaine-binding aptamer. A combined NMR and calorimetry study. Biochemistry.

[31] Shoara AA (2018). Development of a thermal-stable structure-switching cocaine-binding aptamer. Biochimie.

[32] Shoara AA, Churcher ZR, Steele TWJ, Johnson PE (2020). Analysis of the role played by ligand-induced folding of the cocaine-binding aptamer in the photochrome aptamer switch assay. Talanta.

[33] Aviv T (2006). The NMR and X-ray structures of the *Saccharomyces cerevisiae* Vts1 SAM domain define a surface for the recognition of RNA hairpins. J. Mol. Biol..

[34] Slavkovic S, Johnson PE (2018). Isothermal titration calorimetry studies of aptamer-small molecule interctions: Practicalaties and pitfalls. Aptamers.

[35] Freiburger LA, Auclair K, Mittermaier AK (2009). Elucidating protein binding mechanisms by variable-c ITC. ChemBioChem.

[36] Churcher ZR, Johnson PE (2020). NMR for non-experts; a practical guide for applying NMR methods in studies of aptamer-ligand interactions. Aptamers.

