# Supplementary material for: DNA binding by the antimalarial compound artemisinin
Source: Sci Rep. 2022 Jan 7;12:133. doi: 10.1038/s41598-021-03958-6 (PMC8741894; doi:10.1038/s41598-021-03958-6)
Supplement: Supplementary file 1 — Supplementary Figures. [file 41598_2021_3958_MOESM1_ESM.pdf]

Supplementary Data

DNA Binding by the Antimalarial Compound Artemisinin

Sladjana Slavkovic[1], Aron A. Shoara[1], Zachary R. Churcher[1], Elise Daems[2,3,4], Karolien de Wael[3,4], Frank Sobott[2,5,6], Philip E. Johnson[1]*

[1]Department of Chemistry & Centre for Research on Biomolecular Interactions, York University, 4700 Keele St., Toronto, Ontario, Canada, M3J 1P3

[2]BAMS Research group, University of Antwerp, Groenenborgerlaan 171, 2020 Antwerp, Belgium

[3]AXES Research group, University of Antwerp, Groenenborgerlaan 171, 2020 Antwerp, Belgium

[4]Nanolab Centre of Excellence, University of Antwerp, Groenenborgerlaan 171, 2020, Antwerp, Belgium

[5]Astbury Centre for Structural Molecular Biology, University of Leeds, Leeds, LS2 9JT, United Kingdom

[6]School of Molecular and Cellular Biology, University of Leeds, Leeds, LS2 9JT, United Kingdom

*Corresponding author: PEJ - Department of Chemistry, York University, 4700 Keele St., Toronto, Ontario, Canada, M3J 1P3, email: pjohnson@yorku.ca

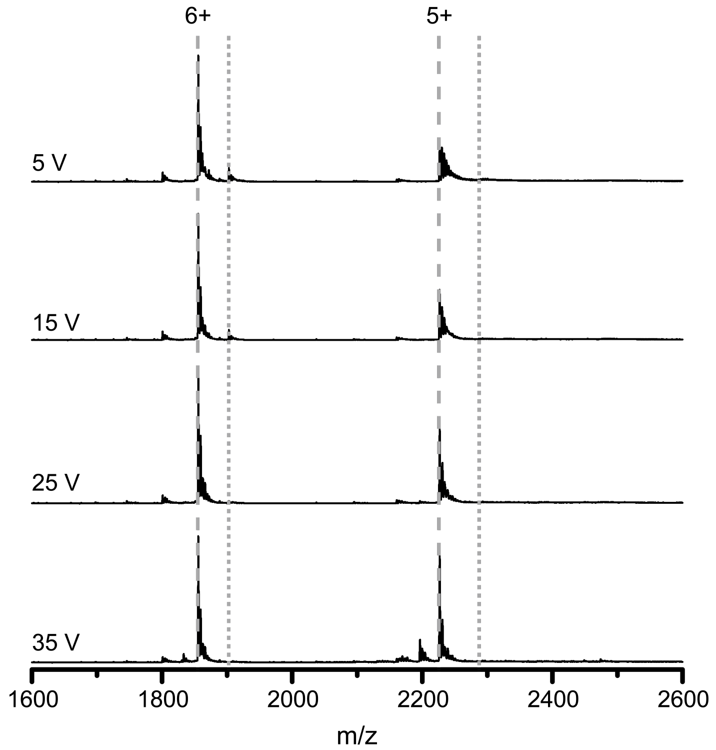

Supplemental Figure 1. Native MS spectra of the MN4 aptamer with artemisinin (1:10 aptamer:artemisinin ratio) in 300 mM ammonium acetate at varying trap collision energies (5-35 V from top to bottom). Theoretical m/z-values of the apo form (dashed lines) and the 1:1 stoichiometry of the complex (dotted lines) are indicated for the 6+ and 5+ charge states.

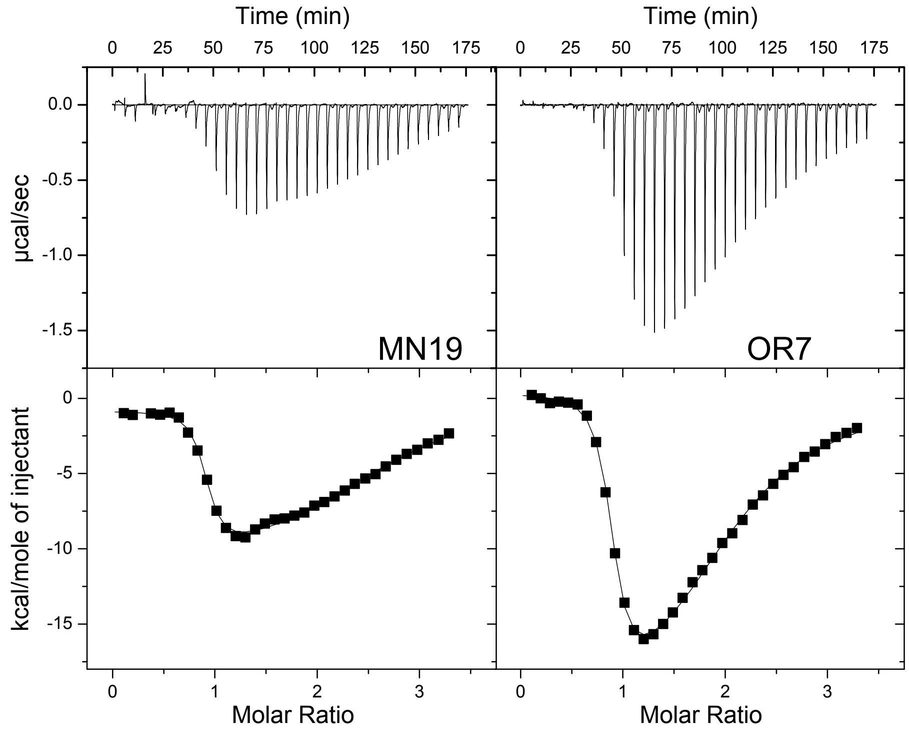

Supplemental Figure 2. ITC thermograms showing interaction of artemisinin with MN19 and OR7 aptamers in buffer containing 20 mM TRIS (pH 7.4), 140 mM, 5 mM KCl, 2.3% (v/v) DMSO. On top is the raw titration data showing the heat resulting from each injection of ligand into aptamer solution. The bottom shows the integrated heat plot after correcting for the heat of dilution.

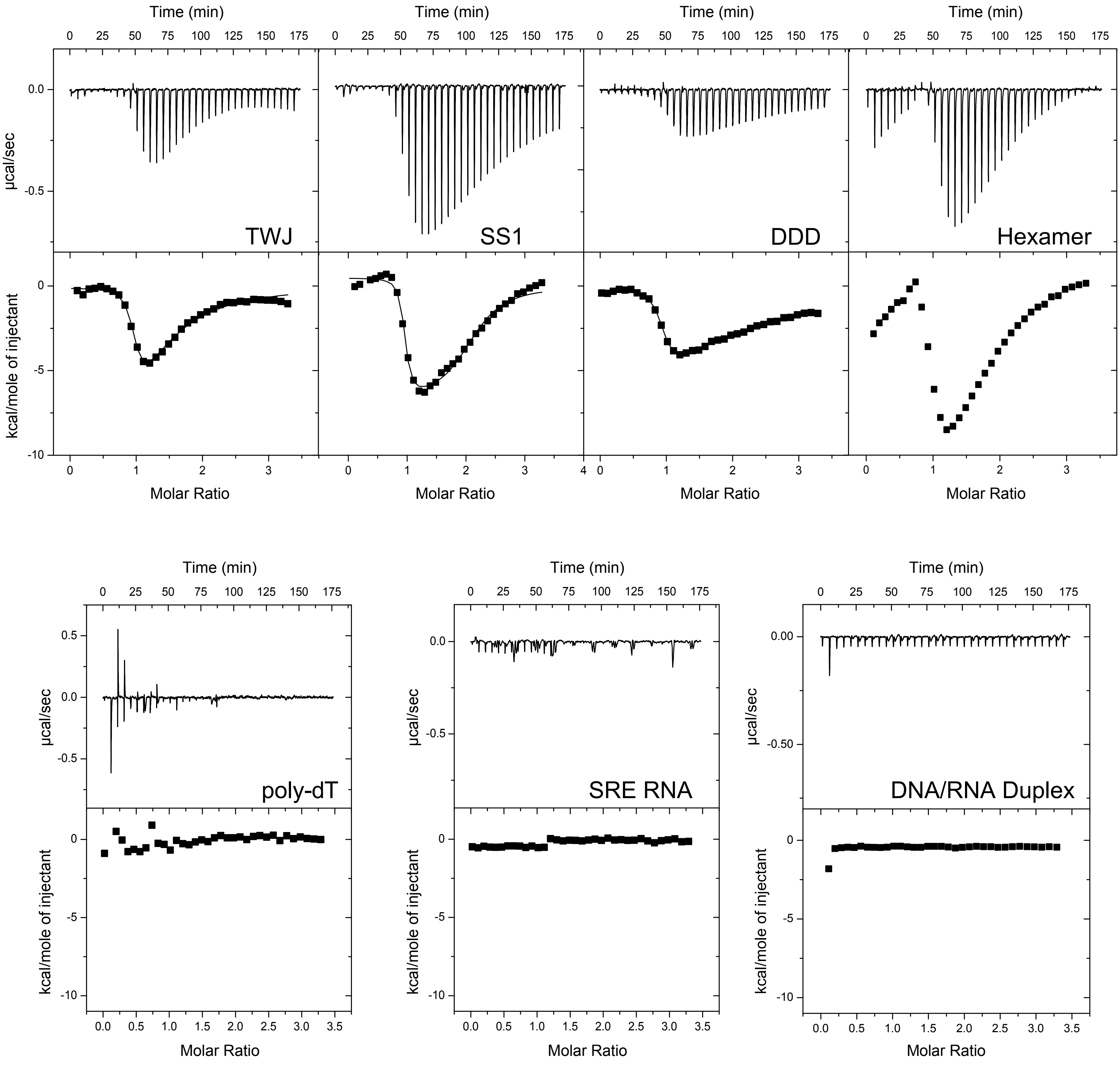

Supplemental Figure 3. ITC thermograms showing interaction of artemisinin with various constructs in buffer containing 20 mM TRIS (pH 7.4), 140 mM, 5 mM KCl, 2% (v/v) DMSO. On top is the raw titration data showing the heat resulting from each injection of ligand into aptamer solution. The bottom shows the integrated heat plot after correcting for the heat of dilution.

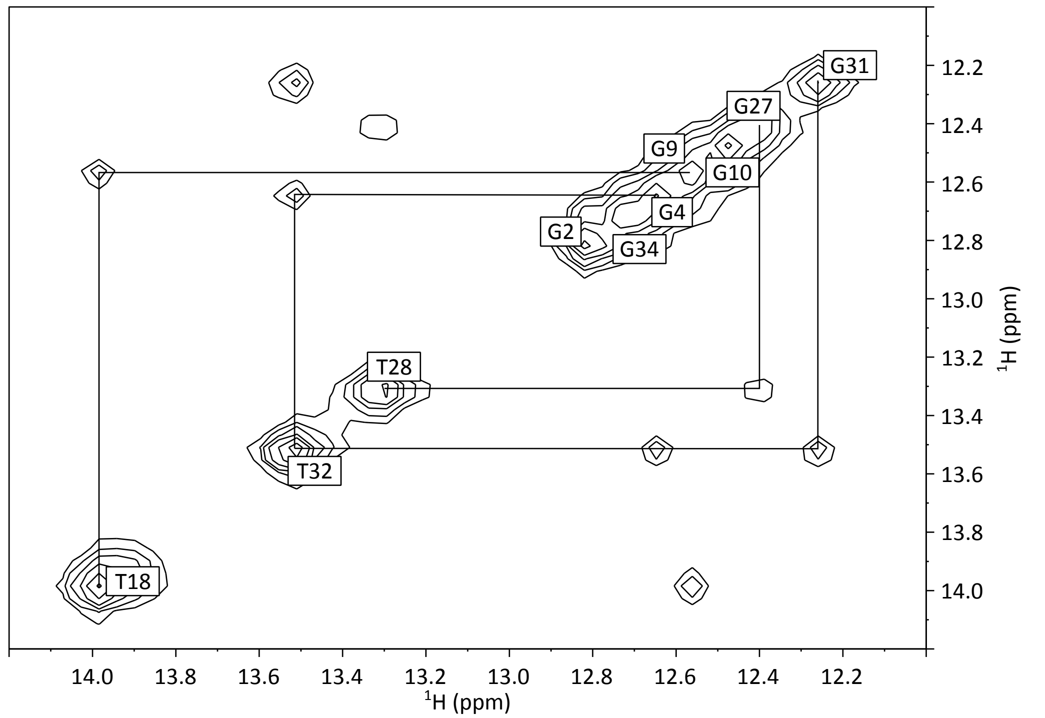

Supplemental Figure 4. ^{1}H-^{1}H NOESY showing the imino-imino cross peak of a sample of MN4 bound to artemisinin. Sample contained 1.4mM aptamer, 20mM $H_xNa_yPO_4$, pH 7.4, 10% D_2O. Sample contains ~3% DMSO-d_6. Spectra acquired at 5 °C.

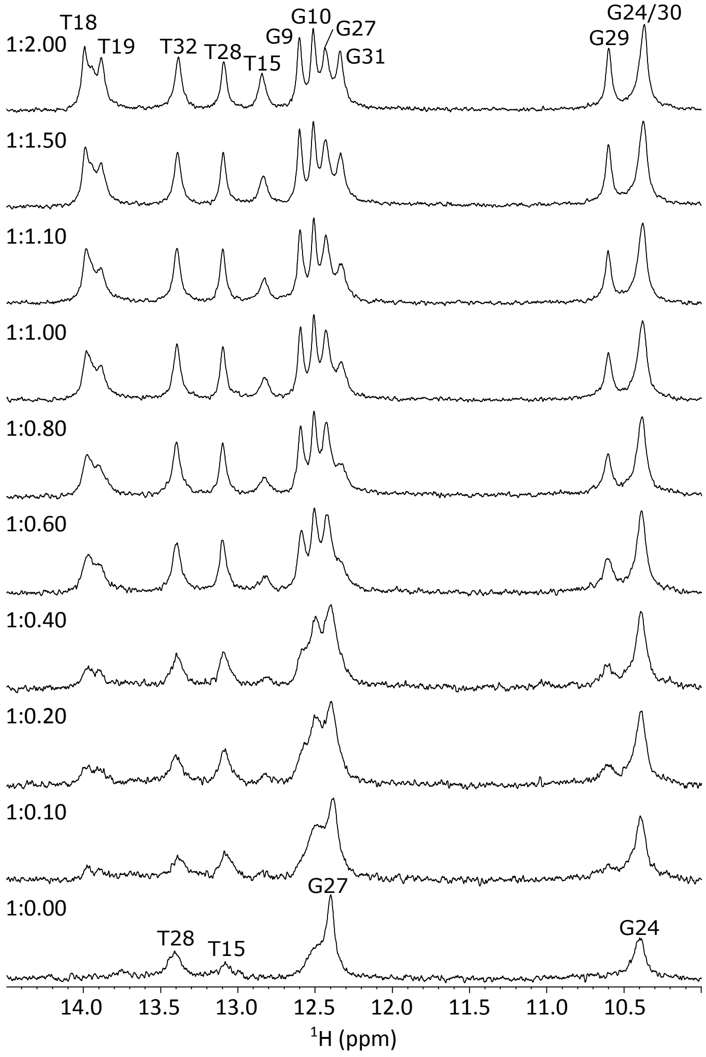

Supplemental Figure 5. ^{1}H NMR spectra showing the imino proton resonances of MN19 as artemisinin is titrated into the sample up to a 1:2.0 molar ratio of aptamer to ligand. Sample contained 400 μM aptamer, 20mM $H_xNa_yPO_4$, pH 7.4, 10% D_2O. Final titration point contains ~3% DMSO-d_6. Spectra acquired at 5 °C

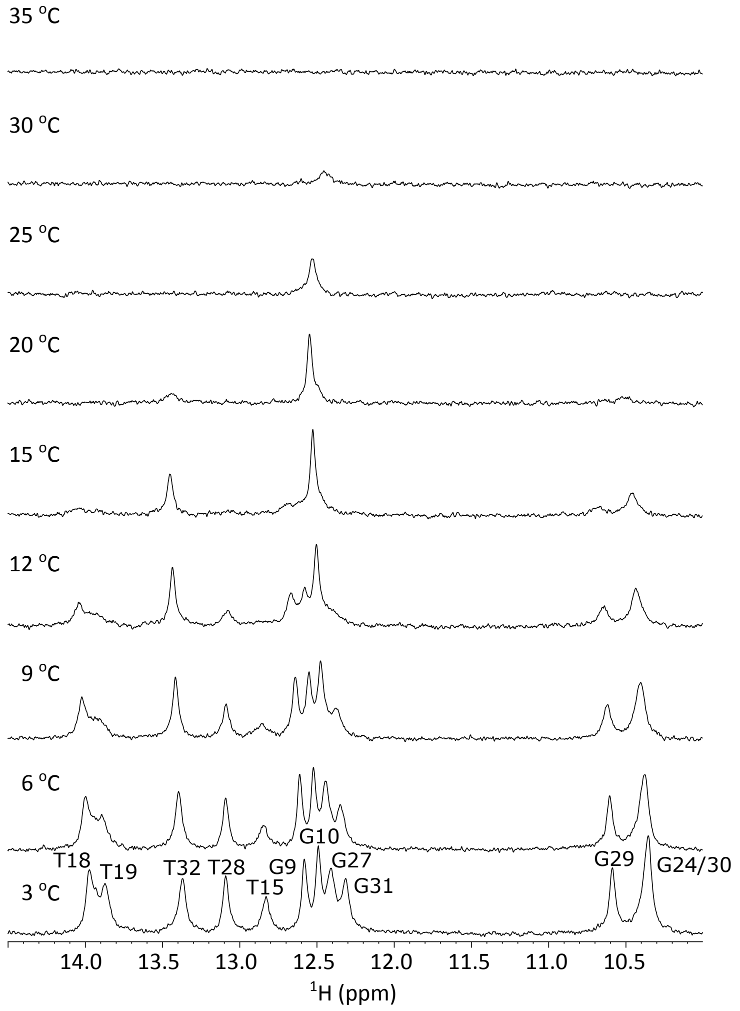

Supplemental Figure 6. ^{1}H NMR spectra showing the imino proton resonances of a temperature scan of MN19 bound to artemisinin. Temperatures ranged from 3 °C to 35 °C. Sample contained 400 μM aptamer, 20 mM $H_xNa_yPO_4$, pH 7.4, 10% D_2O. Sample contains ~3% D6-DMSO.

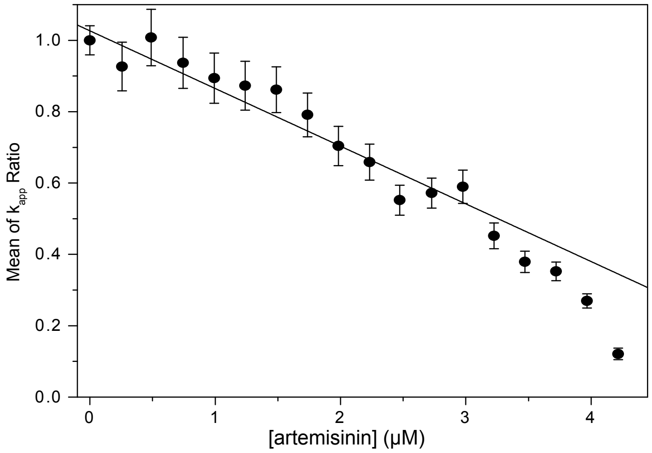

Supplemental Figure 7. exhibits a calibration plot for the normalized average kapp values of MN19-SITS against artemisinin concentrations. The obtained linear slope = (-1.6 ± 0.13) x 10^{-1} and R^2 = 0.96. Triplicated experiments were performed in 20 mM Tris (pH 7.4), 140 mM NaCl at 20 °C. The error bars correspond to one standard deviation
